# The effect of community intervention based on deep learning-established early screening system and model construction for stroke

**DOI:** 10.1186/s12883-026-05127-6

**Published:** 2026-07-14

**Authors:** Xiaowei Chen, Meijun Zhang, Liping Ni, Chunmei Lu, Ang Lu

**Affiliations:** Dongyang Traditional Chinese Medicine Hospital, Dongyang, Zhejiang 322100 China

**Keywords:** Individualized intervention, Machine learning, Mass screening, Risk prediction, Stroke

## Abstract

**Background:**

Stroke is a common cardiovascular complication. The machine learning (ML) algorithms in stroke is a new and noble approach. The objective of the study is to study the practicability of establishing a community-based stroke risk exploratory feature identification using ML and evaluate the effectiveness of individualized interventions in preventing stroke.

**Methods:**

Retrospective data was retrieved of stroke patients (*n* = 158) and healthy population (*n* = 358) who underwent physical examinations in the community. Random forest and Least Absolute Shrinkage and Selection Operator (LASSO) regression were chosen as feature variables. To create a model for predicting the risk of stroke, the multivariate logistic regression analysis was used. Then, a prospective study was conducted with 72 high-risk individuals and divided randomly. Research group received tailored interventions and conventional interventions, while the control group received routine interventions.

**Results:**

Triglycerides, apolipoprotein B (apoB), serum creatinine, high sensitivity C-reactive protein (hsCRP), and homocysteine were determined as risk factors for stroke by the exploratory feature identification. The prospective study revealed no statistically significant differences in baseline characteristics between the two groups (*P*>0.05). The incidence of stroke in research group was 5.41%, remarkably lower than the control group (28.6%, *P*>0.05). The time to stroke occurrence was significantly longer in research group than in control group (*P*>0.05).

**Conclusion:**

The community-based stroke risk exploratory feature identification established using ML can effectively screen high-risk populations. Individualized intervention measures can reduce the incidence of stroke significantly.

## Background

Stroke is a prevalent and severe illness that poses a major risk to human health and is characterized by high incidence, disability, and death. More than 15 million new stroke cases occur worldwide each year, with approximately 6 million deaths, and among survivors, around 50% are left with varying degrees of functional impairment [1]. In China, stroke is the major cause of death and disability among adults, putting a significant strain on families of patients and society overall [2]. So, early identification of risk groups and the implementation of focused preventive actions are crucial for reducing stroke risk and improving outcomes.

Traditional stroke risk assessments often rely on empirical models such as the Framingham Risk Score. However, their predictive effectiveness is influenced by population and regional differences, making it challenging to meet the needs of precise prevention [3–5]. The ML has been used extensively in the medical industry in recent years, providing novel methods for predicting disease risk [6]. Prediction models based on ML algorithms can effectively integrate multidimensional clinical data, uncover hidden patterns, and improve the accuracy and personalization of prediction of stroke risk as depicted by several studies [7–10]. However, research focusing on building stroke risk prediction models for community population is relatively scarce as most existing studies are conducted in hospital populations.

Furthermore, there is still a pressing need to develop tailored intervention techniques and use these predictive models to direct community stroke prevention and control initiatives. Prior research has demonstrated that stroke risk can be successfully decreased by pharmacological therapies and lifestyle modifications that target high-risk variables [11]. But conventional intervention models frequently employ a “one-size-fits-all” approach that ignores individual differences, leading to less than ideal intervention results [12]. It is possible to achieve accurate prevention and control, increase intervention efficiency, and improve adherence by combining risk prediction with tailored therapies.

This study aims to apply ML algorithms to establish a stroke risk exploratory feature identification in a community population and, based on this model, explore the preventive effects of individualized interventions for high-risk groups. The research results are expected to provide new ideas and practical experiences for community-based stroke prevention and control, with significant theoretical and practical value.

## Methods

### Ethical considerations

The study was approved by the Institutional Review Board (or Ethics Committee) of Dongyang Traditional Chinese Medicine Hospital with acceptance number 2,502,240,842,427 on 2025/2/27. The subjects were explained in detail about the study and allowed to ask questions. Those subjects who consented were included, and written informed consent to participate was obtained from all of the participants.

### Study design

The study utilized two methods: Phase 1: Retrospective cohort for model development, Phase 2: Prospective randomized controlled trial for intervention evaluation.

### Study population

#### Retrospective collection

This study retrospectively collected data from patients diagnosed with stroke who visited community medical institutions in the city between 2019 and 2021. Stroke cases were identified from medical records of community medical institutions and were confirmed based on clinical diagnosis together with neuroimaging findings on computed tomography or magnetic resonance imaging. For participants in the control group, the absence of stroke was verified from contemporaneous health examination records and medical history. Simultaneously, a control group was selected from individuals who underwent routine health check-ups in the same communities during the same period, and were screened as not having stroke or other severe diseases.

Inclusion criteria: ① Community residents; ② Age ≥ 18 years; ③ Complete medical records, including demographic information, laboratory parameters, imaging characteristics, and clinical history; ④ The most recent follow-up date was at least one year after the baseline data collection.

Exclusion criteria: ① Presence of severe diseases such as malignant tumors; ② Missing or misleading data; ③ Loss to follow-up.

#### Prospective collection

Using the stroke risk exploratory feature identification established in Sect.  1.1, individuals in the community were screened for high-risk stroke groups. The participants from high-risk population was chosen for the study using a stratified sample technique. The randomization opted was permuted block based. Inclusion criteria: ① Community residents; ② Age ≥ 18 years; ③ Stroke risk > 50% as assessed by the risk exploratory feature identification (The threshold is acknowledged as exploratory as it is a part of a pilot design); ④ Voluntarily providing informed consent.

Exclusion criteria: ① Presence of severe diseases such as malignant tumors; ② History of stroke; ③ Expected survival of less than one year; ④ Loss to follow-up, etc.

### Intervention measures

A schematic diagram illustrates the community-based approaches implemented.

### Control group

1. Stratify the population based on the “8 + 2” risk exposure factors: [12] 1) High-risk distribution: Those with ≥ 3 risk factors are categorized into the “≥3 risk factors” group, and those with a history of Transient Ischemic Attack (TIA).
2. or stroke are placed in the “TIA/Stroke” group. 2)Medium-risk distribution: Individuals with < 3 risk factors but with chronic conditions such as hypertension, diabetes, atrial fibrillation, or valvular heart disease. 3)General community population: Those with ≤ 2 risk factors and with no chronic diseases.

(2) Different interventions based on stratification results: 1) For the general community population: (a) Standardized training for community doctors to guide patients in lifestyle changes and control risk factors (blood pressure, cholesterol, and blood sugar) according to relevant guidelines; (b) During stroke screening, promote knowledge about stroke risk factors and preventive measures; (c) Regularly post stroke prevention posters and distribute relevant educational materials in the community; (d) Organize periodic free clinics in the community, providing medication guidance and stroke prevention knowledge, aiming for at least three sessions per year. 2) For the medium/high-risk community population: (a) Same measures as for the general population; (b) Perform carotid ultrasound during stroke screening and provide lifestyle guidance and medication advice through medical visits; (c) Hold at least three stroke prevention seminars annually.

### Study group: individualized intervention based on the exploratory feature identification

It was a pilot exploratory phase of the study with limited power. In this study, the stroke risk exploratory feature identification was applied to perform individualized risk assessments for the experimental group. High-risk factors such as triglycerides, apoB, serum creatinine, hsCRP, and homocysteine were identified, and targeted interventions were implemented accordingly.

**Table:** 

High-Risk Factor	Intervention Measures
Triglycerides	1. Dietary intervention: Limit intake of high-sugar and high-fat foods, increase the intake of dietary fiber and unsaturated fatty acids. 2. Exercise intervention: Develop personalized exercise prescriptions, such as brisk walking, swimming, and moderate-intensity aerobic exercise per week of 150–300 min. 3. If necessary, provide fibrate medication and regularly monitor triglyceride levels.
ApoB	1. Dietary intervention: Limit intake of saturated and trans fats, promote the Mediterranean diet pattern. 2. Exercise intervention: Same as for triglycerides. 3. If necessary, provide statin medication and regularly monitor apolipoprotein B levels.
Serum Creatinine	1. Blood pressure control: Combine lifestyle interventions with medication to maintain blood pressure below 130/80 mmHg. 2. Protein intake restriction: Control protein intake to 0.8 g/(kg·d), prioritizing high-quality proteins. 3. Regularly monitor kidney function and adjust treatment according to the stage of kidney disease.
Hs CRP	1. Anti-inflammatory treatment: Administer antiplatelet drugs such as aspirin, add statins if necessary. 2. Infection control: Timely diagnosis and treatment of infectious diseases such as periodontitis and pneumonia. 3. Improvement of insulin resistance: Comprehensive interventions through diet, exercise, and medication to improve insulin sensitivity.
Homocysteine	1. Supplementation of Vitamin B: Daily supplementation of 10–25 mg vitamin B6, 500–1000 µg vitamin B12, and 0.4-1.0 mg folic acid. 2. Restriction of methionine intake: Moderately reduce meat intake and increase the proportion of fresh fruits and vegetables. 3. Regularly monitor blood homocysteine level, and if needed, prescribe pyridoxine or other medications.

All participants in the experimental group had undergone follow-up every two months to evaluate the compliance to the intervention plans and changes in clinical indicators. The customized intervention measures will be modified as needed. Health education will also be reinforced to enhance participants’ awareness of stroke risk factors and to improve their ability to actively manage their health.

### Collection of observation indicators

#### Collection of demographic data for both patient groups

Demographic information: Gender, age, and body mass index; Laboratory tests: Diastolic blood pressure, systolic blood pressure, white blood cells, total cholesterol, lipoproteins, apolipoprotein A, apolipoprotein B, triglycerides, high-density lipoprotein, low-density lipoprotein, serum creatinine, high-sensitivity C-reactive protein, fibrinogen, and homocysteine; Clinical history: Hypertension, diabetes, heart disease, and atherosclerosis/stroke/occlusion.

#### 12-month follow-up for both groups

The primary outcome during the 12-month follow-up was the occurrence of a first stroke event. Suspected stroke events were identified during scheduled follow-up and were verified by review of clinical records, physician diagnosis, discharge documentation, and neuroimaging findings on computed tomography or magnetic resonance imaging when available. The date of the first confirmed stroke event was recorded for outcome analysis. Outcome adjudication was not formally blinded to group allocation.

### Statistical analysis procedure

R software (version 4.1.0) was used to conduct the statistical analysis. Independent sample t-tests or Mann-Whitney U tests were used for group comparisons, and the mean ± standard deviation (Mean ± SD) or median (interquartile range) [median (Q1, Q3)] are used to display continuous variables. Groups were compared using the chi-square test or Fisher’s exact test, and frequency (percentage) was used to represent categorical variables. To deal with missing data, several imputation techniques were applied. Every statistical analysis was two-tailed, and a *p* < 0.05 was considered statistically significant. The stroke risk exploratory feature identification’s features were chosen using ML techniques like LASSO regression and random forest [13]. The independent risk factors for stroke were identified using multivariable logistic regression analysis after shared variables were identified using a Venn diagram. Internal validation was done and a nomogram was used to assess the model’s predictive performance. In the prospective study phase, baseline data for the study and control groups were analyzed for intergroup differences using t-tests or Mann-Whitney U tests for continuous variables and chi-square tests or Fisher’s exact tests for categorical variables. The outcome assessors were blinded to group assignment. The differences in stroke incidence between the two groups were compared using a chi-square test. The time to stroke occurrence for the two groups was compared using Log-rank tests and K-M survival curves. The potential confounders were socioeconomic status of the subjects; medication use and health behaviors such as physical exercise and diet. The limitation of the study is that these confounders weren’t adjusted.

## Results

### Community stroke risk prediction mod exploratory feature identification el established using ML

A total of 158 stroke patients diagnosed at various community medical institutions in the city from 2019 to 2021 were selected as the study group, while 378 individuals who underwent regular health checkups at the same period in the city’s communities were selected as the control group (Fig. 1). Demographic data, laboratory test and clinical history were collected for both patient groups. The comparison of data between the two groups is shown in (Table 1). Based on these indicator data, feature variables were selected using LASSO regression (Figs. 2A, B) and random forest (Fig. 2C). A Venn diagram was used to identify five shared variables (Fig. 2D). Multivariable regression analysis was conducted based on these five shared variables, and the results revealed that triglycerides, apoB, serum creatinine, hsCRP, and homocysteine are independent risk factors for stroke (*P* < 0.05, Fig. 3A). A nomogram was created based on these five factors to establish a stroke risk exploratory feature identification for patient screening (Fig. 3B).

**Table 1:** Comparison of clinical data between stroke patients and healthy volunteers

	[ALL]	Healthy Control Group	Stroke Group	*P* value
	*N* = 536	*N* = 378	*N* = 158	
Age	47.6 ± 8.72	45.4 ± 8.08	52.9 ± 7.95	< 0.0001
Gender				
Female	248(46.3%)	186(49.2%)	62(39.2%)	0.0439
Male	288(53.7%)	192(50.8%)	96(60.8%)	
Body Mass Index (BMI)	32.4 ± 5.03	31.9 ± 4.62	33.6 ± 5.73	7e-04
Hypertension				
0	337(62.9%)	284(75.1%)	53(33.5%)	< 0.0001
1	199(37.1%)	94(24.9%)	105(66.5%)	
Diabetes				
0	457(85.3%)	350(92.6%)	107(67.7%)	< 0.0001
1	79(14.7%)	28(7.41%)	51(32.3%)	
Heart Disease				
0	501(93.5%)	366(96.8%)	135(85.4%)	< 0.0001
1	35(6.53%)	12(3.17%)	23(14.6%)	
Arteriosclerosis				
0	433(80.8%)	340(89.9%)	93(58.9%)	< 0.0001
1	103(19.2%)	38(10.1%)	65(41.1%)	
Systolic BP	139[86.0;211]	135[86.0;186]	154[100;211]	< 0.0001
Diastolic BP	84.6 ± 14.2	84.8 ± 13.2	84.1 ± 16.4	0.6290
Total Cholesterol	4.82[0.04;11.2]	4.52[0.91;9.26]	6.24[0.04;11.2]	< 0.0001
Triglycerides	1.71[0.05;5.81]	1.48[0.05;3.09]	3.00[0.24;5.81]	< 0.0001
High Density Lipoprotein Cholesterol (HDL-C)	1.42 ± 0.40	1.51 ± 0.40	1.20 ± 0.30	< 0.0001
Low Density Lipoprotein Cholesterol (LDL-C)	1.97[0.63;3.44]	1.96[0.63;3.34]	2.08[0.96;3.44]	0.0358
Apolipoprotein A (ApoA)	1.59[0.25;2.78]	1.60[0.25;2.78]	1.58[0.80;2.54]	0.5622
Apo B	0.94[0.01;2.13]	0.80[0.01;2.09]	1.50[0.50;2.13]	< 0.0001
Serum Creatinine	74.7[13.2;140]	64.8[13.2;120]	99.1[46.4;140]	< 0.0001
hsCRP	11.4[0.27;47.4]	9.71[0.27;18.6]	24.8[1.20;47.4]	< 0.0001
Fibrinogen	3.65[0.03;11.3]	3.36[0.03;7.16]	5.02[0.03;11.3]	< 0.0001
Homocysteine	11.1[0.05;31.7]	9.94[0.05;18.1]	16.8[0.16;31.7]	< 0.0001

**Fig. 1:**
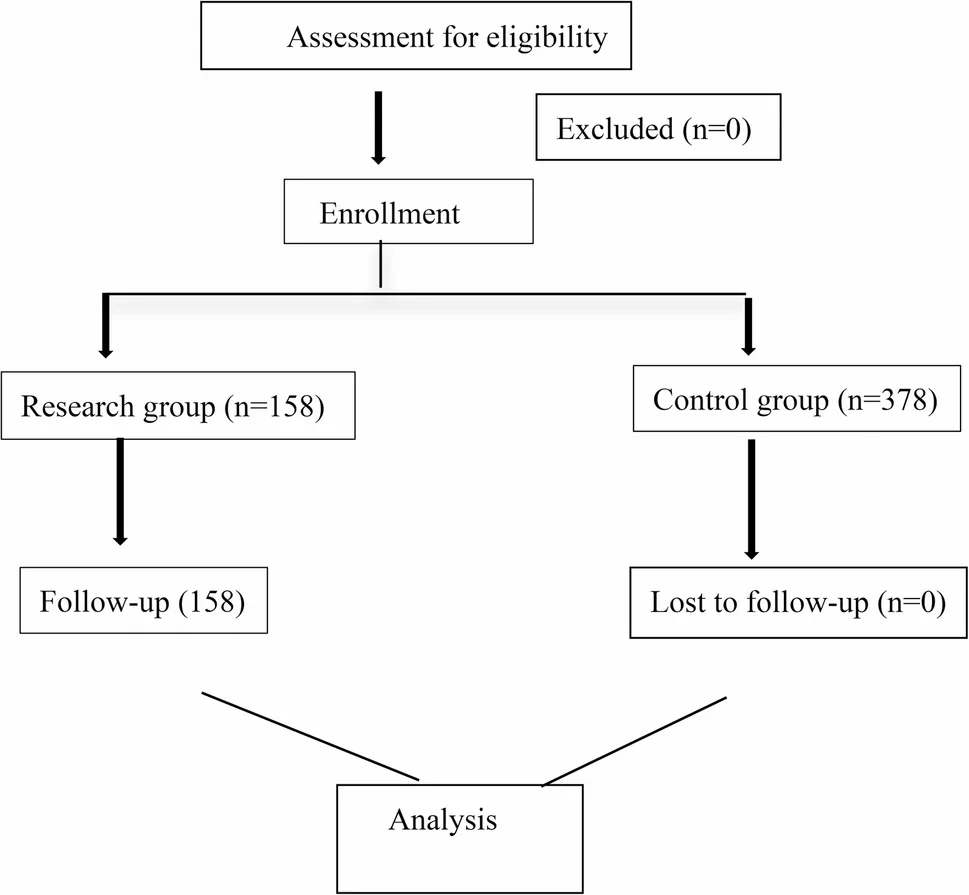
Flow diagram depicting subject enrollment

**Fig. 2:**
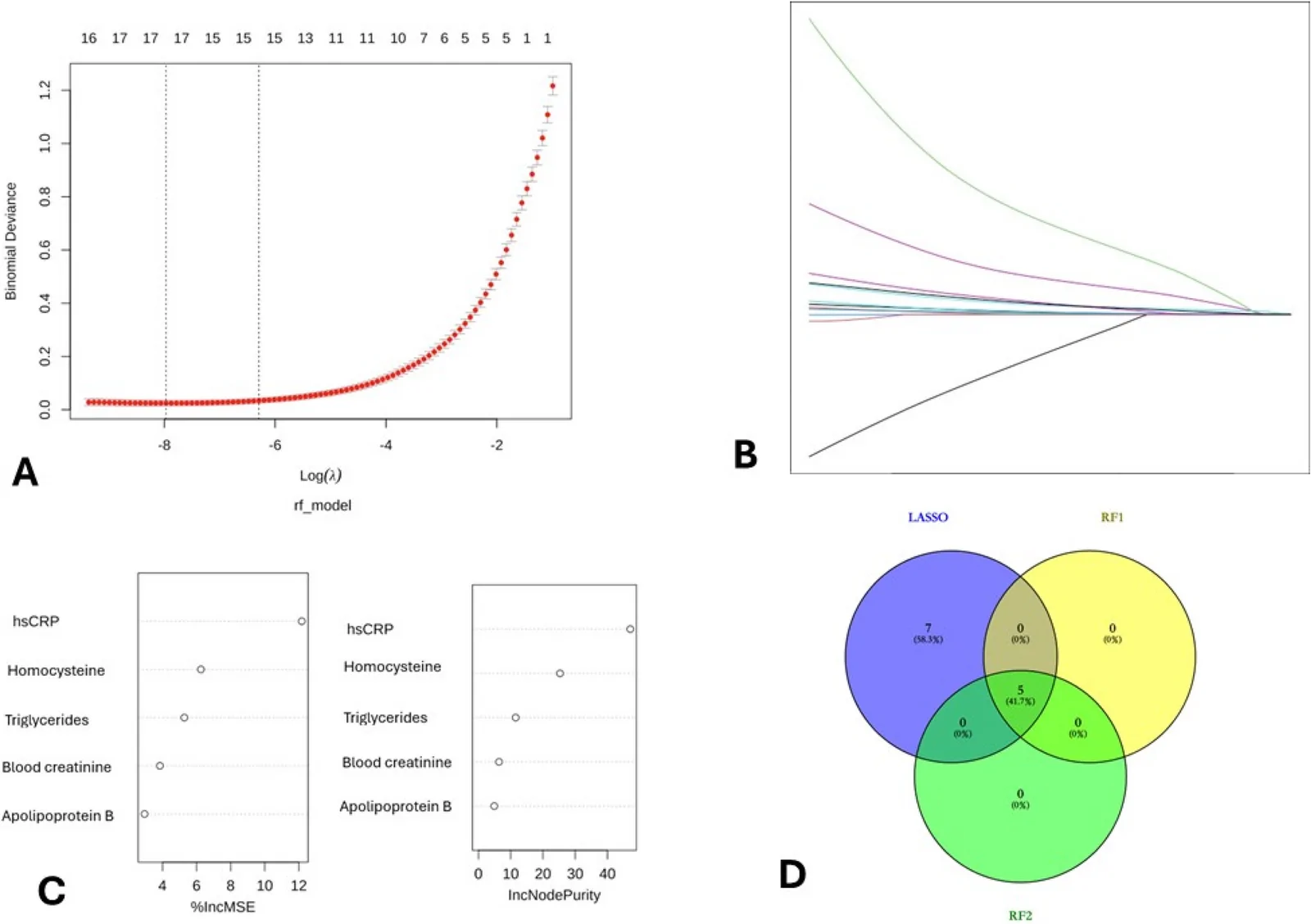
Core variables for stroke patients based on ML **A**, **B**. LASSO regression; **C**. Random forest; **D**. Venn diagram

**Fig. 3:**
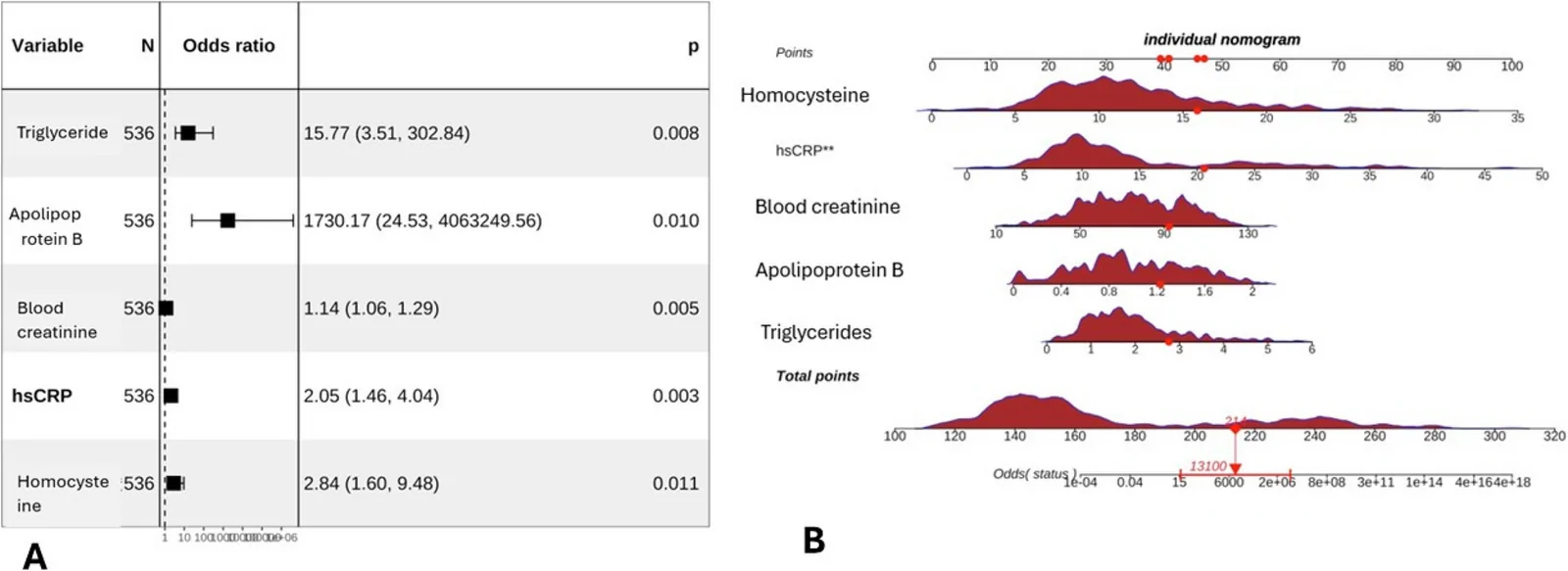
Risk exploratory feature identification for screening stroke patients based on regression analysis. **A** Forest plot showing the results of multivariable regression analysis; **B**. Nomogram demonstrating the risk exploratory feature identification for screening stroke patients

### Comparison of baseline data of prospectively enrolled patients

A total of 72 patients were enrolled in this study, based on the inclusion and exclusion criteria. All were evaluated as high-risk (with a stroke occurrence probability > 50%), according to the risk exploratory feature identification. Among them, 35 patients were in the control group, and 37 patients were in the study group. Clinical data results for both groups are shown in (Table 2). There were no significant statistical differences (*P* > 0.05) between the two groups in terms of demographic data (gender, age, body mass index), laboratory test indicators (systolic pressure, diastolic pressure, white blood cell count, high-density lipoprotein, low-density lipoprotein, lipoproteins, total cholesterol, triglycerides, apoA, apoB, serum creatinine, hsCRP, fibrinogen, and homocysteine), and clinical history (hypertension, diabetes, heart disease, arteriosclerosis/narrowing/occlusion), as indicated by the results.

**Table 2:** Comparison of baseline characteristics between two patient groups

	[ALL]	Control Group	Study Group	*P* value
	*N* = 72	*N* = 35	*N* = 37	
Age	52.9 ± 7.56	53.1 ± 7.45	52.6 ± 7.77	0.7843
Gender				0.9682
Female	30(41.7%)	14(40.0%)	16(43.2%)	
Male	42(58.3%)	21(60.0%)	21(56.8%)	
BMI	33.3 ± 5.58	32.1 ± 6.07	34.4 ± 4.89	0.0755
Hypertension				0.3493
0	20(27.8%)	12(34.3%)	8(21.6%)	
1	52(72.2%)	23(65.7%)	29(78.4%)	
Diabetes				0.6937
0	53(73.6%)	27(77.1%)	26(70.3%)	
1	19(26.4%)	8(22.9%)	11(29.7%)	
Heart Disease				1.0000
0	63(87.5%)	31(88.6%)	32(86.5%)	
1	9(12.5%)	4(11.4%)	5(13.5%)	
Arteriosclerosis				0.3609
0	44(61.1%)	19(54.3%)	25(67.6%)	
1	28(38.9%)	16(45.7%)	12(32.4%)	
Systolic Pressure	151 ± 21.5	149 ± 23.1	153 ± 20.0	0.5103
Diastolic Pressure	86.5[40.0;109]	83.0[51.0;109]	92.0[40.0;109]	0.5279
Total Cholesterol	6.33 ± 2.11	6.28 ± 2.37	6.38 ± 1.87	0.8311
Triglycerides	3.06 ± 1.20	3.16 ± 1.12	2.95 ± 1.27	0.4590
HDL	1.20 ± 0.29	1.14 ± 0.29	1.25 ± 0.29	0.0969
LDL	2.10 ± 0.53	2.05 ± 0.51	2.14 ± 0.55	0.4617
ApoA	1.64 ± 0.29	1.67 ± 0.31	1.60 ± 0.28	0.3561
ApoB	1.51 ± 0.31	1.48 ± 0.26	1.54 ± 0.35	0.3825
Serum Creatinine	95.4 ± 15.9	97.5 ± 13.6	93.4 ± 17.8	0.2637
hsCRP	25.3 ± 7.58	25.1 ± 7.81	25.4 ± 7.46	0.8738
Fibrinogen	5.54 ± 2.46	5.34 ± 2.81	5.73 ± 2.09	0.5114
Homocysteine	17.2 ± 5.43	17.2 ± 5.70	17.1 ± 5.23	0.9457

### Study on the preventive effect of different interventions on stroke incidence in patients

The average follow-up time for both groups of patients was 9.93 ± 3.15 months. Among the control group, 10 out of 35 patients suffered from stroke, with an incidence rate of 28.6% (10/35). In the study group, 2 out of 37 patients were diagnosed with stroke, with an incidence rate of 5.41% (2/37). As compared to the control group, the study group’s stroke incidence rate was significantly lower (*P* < 0.05). The findings are depicted in (Table 3).

The Kaplan–Meier (K-M) survival analysis was performed with stroke incidence as the outcome event. The results revealed that the median time to stroke occurrence for the 72 patients was 16.9 months (*95% CI: 13.5-NA*). Among them, the control group had a median stroke occurrence time of 16.9 months (*95% CI: 10.2-NA*), while the study group had NA (*95% CI: NA-NA*). The difference was statistically significant (*P* < 0.05). The results are shown in (Fig. 4; Table 4).

**Table 3:** Comparison of stroke incidence between two patient groups

	[ALL]	Control Group	Study Group	*P* value
	*N* = 72	*N* = 35	*N* = 37	
Stroke Incidence:				
0	60(83.3%)	25(71.4%)	35(94.6%)	0.0203
1	12(16.7%)	10(28.6%)	2(5.41%)	

**Table 4:** K-M analysis of stroke incidence between two patient groups

	*n*	events	median	0.95LCL	0.95UCL
Control Group	35	10	16.9	10.2	NA
Study Group	37	2	NA	NA	NA
Total	72	12	16.9	13.5	NA

**Fig. 4:**
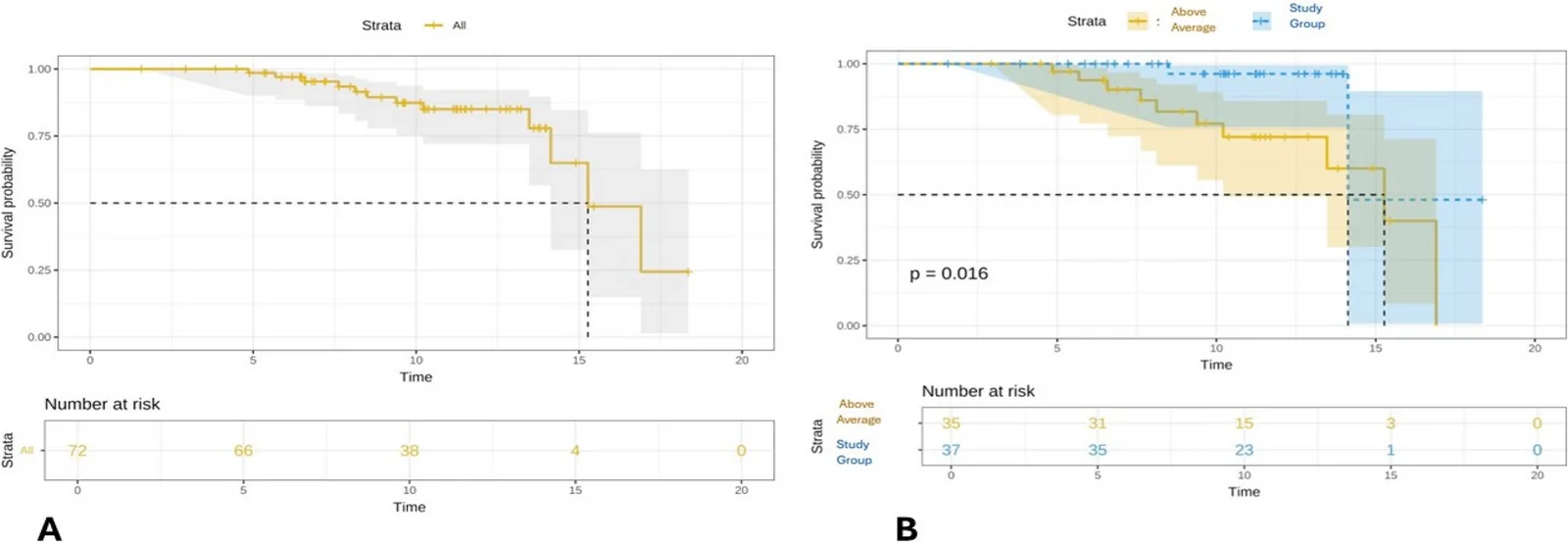
K-M curves comparing the incidence of stroke. **A** Overall K-M analysis (**B**) Comparison of K-M curves between two patient groups

## Discussion

### Feasibility and effectiveness of the community stroke risk exploratory feature identification ased on ML

This study is based on retrospective clinical data collected from community stroke patients and healthy control population. Through ML algorithms such as LASSO regression and random forests, we identified five common variables—triglycerides, apoB, serum creatinine, hsCRP, and homocysteine—and further confirmed them as independent risk factors for stroke through multivariate regression analysis. These findings are consistent with previous research reports [13]. Meta-analysis has shown that hypertriglyceridemia is an independent risk factor for ischemic stroke, with a 27% increase in the risk of ischemic stroke for every 1 mmol/L increase in triglycerides [14, 15]. The apoB is a direct promoter of atherosclerosis, and cohort studies have shown a positive correlation between apoB levels and ischemic stroke risk. After adjusting for other factors, for every 15 mg/dL increase in apoB, the risk of ischemic stroke increases by 6% [16]. Furthermore, there is a strong correlation between apolipoprotein B and the incidence of stroke, according to MR analysis [17]. Chronic kidney disease (CKD) is an independent risk factor for stroke, and serum creatinine is crucial for evaluating renal function. According to a meta-analysis, eCKD is linked to a 1.31 (95% CI: 1.21–1.43) hazard ratio (HR) for stroke [18]. The development of atherosclerosis is significantly influenced by inflammation, and hsCRP is a sensitive indicator of inflammation. Participants with hsCRP ≥ 3.0 mg/L have a 64% higher risk of ischemic stroke than those with hsCRP < 1.0 mg/L [19]. Elevated homocysteine causes endothelial damage and promotes atherosclerosis.

A research has shown that the stroke recurrence rate is 13.6% among hypertensive patients with high homocysteine levels. The risk of stroke recurrence is 1.551 times more than that in patients with only hypertension (*P* < 0.001, 95% CI: 1.315–1.829), and 1.625 times raised than in the normal subjects (*P* < 0.001, 95% CI: 1.357–1.947), after adjusting for other influencing factors [20]. The five risk factors included in this study cover multiple pathological links involved in stroke occurrence, including lipid metabolism disorders, renal dysfunction, inflammation, and endothelial damage. Therefore, the stroke risk exploratory feature identification based on these five factors can comprehensively assess stroke risk in the community population. Furthermore, the LASSO regression and random forests used in this study are mature ML algorithms that can effectively handle high-dimensional data and explore the inherent relationships between variables. Thus, using them to establish the stroke risk exploratory feature identification is feasible. The nomogram risk exploratory feature identification established in this study is simple to use and suitable for community-based implementation.

The study has implications for community-based stroke screening and prevention. The intricate relationships between biochemical, metabolic, and inflammatory parameters may not be sufficiently captured by traditional screening approaches in community settings, which frequently depend on broad clinical evaluations or generalised risk scores. By combining several clinical variables and using data-driven feature selection to find the most significant predictor, the ML-based exploratory feature identification is useful. This method makes it possible to stratify risks more precisely based on the features of local populations. The addition of creatinine and apoB emphasises the need of evaluating lipid-related atherosclerotic load and renal function, which are occasionally overlooked in routine community screening procedures. By including these parameters into screening procedures, metabolic problems and silent vascular damage may be detected earlier.

In summary, this study manifested the feasibility of applying ML in community stroke risk prediction, providing new insights for precise screening of high-risk populations and early intervention.

### Basis and implementation of effectiveness of personalized intervention measures based on risk exploratory feature identification

This study identified high-risk population in the community based on the established stroke risk exploratory feature identification and formulated personalized intervention measures targeting five risk factors—triglycerides, apoB, serum creatinine, hsCRP, and homocysteine. These interventions included comprehensive management in areas such as diet, exercise, medication, and lifestyle. This approach aligns with the recommendations of stroke secondary prevention guidelines as explained in the table above [21–23]. This study further combines individualized risk assessment results to formulate targeted interventions, enhancing the precision and effectiveness of the interventions.

This study prospectively evaluated the implementation effects of individualized interventions, with results showing a significantly lower stroke incidence in the intervention group (5.41% vs. 28.6%) within 12 months. This suggests that individualized interventions can effectively reduce the stroke incidence in high-risk community populations. These findings are consistent with previous studies. For example, a randomized controlled trial involving 2,471 elderly community residents manifested that stroke incidence in the intervention category with individualized management was 2.0%, remarkably lower than 3.8% in the conventional management control category [24]. Another cohort study involving 3,304 high-risk community residents showed that after six years of individualized management, the risk of new stroke in the intervention group was reduced by 26% in comparison to the un-managed group [25]. These findings support the utility of applying personalized comprehensive management strategies in community stroke primary prevention.

This study also looked at the time of stroke occurrence, and it was found that the intervention group experienced a significantly longer period than the control group. This implies that tailored therapies not only lower the incidence of stroke but also postpone its occurrence, which is an important evidence for enhancing patient outcomes. In summary, this study offers crucial evidence for improving community stroke management models and demonstrates that individualized interventions based on the risk exploratory feature identification can successfully prevent stroke occurrence in high-risk community groups.

### Limitations and future outlook

The first limitation is that the sample size is relatively less and the study is conducted in a single community population, so further validation is required. Future research should be conducted with large sample sizes and multi-center studies to obtain more universally applicable conclusions. Second, the follow-up period of this study was relatively short, only 12 months, and the long-term intervention effects need further evaluation. Future studies should extend the follow-up period to dynamically assess the intervention effects. Third, this study primarily evaluated the impact of intervention measures on stroke incidence, but the effects on patient quality of life, medical costs, etc., are not studied. Future research should conduct more comprehensive cost-effectiveness analyses to provide evidence for formulating optimized management strategies. The final limitation is that no external validation was performed for generalizability of the data.

## Conclusions

This study established a community stroke risk exploratory feature identification based on ML algorithms, identifying five independent risk factors: homocysteine, triglycerides, apoB, serum creatinine, and hsCRP. The prospective study results outlined that personalized interventions for high-risk populations can potentially benefit in reducing stroke incidence and delay the occurrence of stroke. This research provides new insights and methods for precise stroke prevention and control in communities, with good prospects for widespread application. Based on the pilot exploratory nature of the intervention phase and limited number of the outcome events, the definitive effectiveness cannot be suggested. Future research should focus on increasing sample sizes, extending follow-up periods, and further validating the external applicability of the exploratory feature identification and the long-term effects of personalized interventions.

## Data Availability

The datasets used and/or analyzed during the current study are available from the corresponding author on reasonable request.

## Abbreviations

ApoA: Apolipoprotein A
ApoB: Apolipoprotein B
BMI: Body Mass Index
BP: Blood Pressure
CKD: Chronic Kidney Disease
HDL-C: High-Density Lipoprotein Cholesterol
hsCRP: High-Sensitivity C-Reactive Protein
K-M: Kaplan-Meier
LASSO: Least Absolute Shrinkage and Selection Operator
LDL-C: Low-Density Lipoprotein Cholesterol
ML: Machine Learning
TIA: Transient Ischemic Attack
